# Human Herpesvirus-6 U14 Induces Cell-Cycle Arrest in G2/M Phase by Associating with a Cellular Protein, EDD

**DOI:** 10.1371/journal.pone.0137420

**Published:** 2015-09-04

**Authors:** Junko Mori, Akiko Kawabata, Huamin Tang, Kenjiro Tadagaki, Hiroyuki Mizuguchi, Kazumichi Kuroda, Yasuko Mori

**Affiliations:** 1 Division of Clinical Virology, Kobe University Graduate School of Medicine, Kobe, 6500017, Japan; 2 Department of Immunology, Nanjing Medical University, Nanjing, 210029, China; 3 Department of Biochemistry and Molecular Biology, Graduate School of Medical Science, Kyoto Prefectural University of Medicine, Kyoto, 6028566, Japan; 4 Laboratory of Biochemistry and Molecular Biology, Graduate School of Pharmaceutical Sciences, Osaka University, Osaka, 5650871, Japan; 5 Division of Microbiology, Department of Pathology and Microbiology, Nihon University School of Medicine, Tokyo, 1738610, Japan; 6 Laboratory of Virology and Vaccinology, National Institute of Biomedical Innovation, Osaka, 5670085, Japan; Virginia Commonwealth University, UNITED STATES

## Abstract

The human herpesvirus-6 (HHV-6) infection induces cell-cycle arrest. In this study, we found that the HHV-6-encoded U14 protein induced cell-cycle arrest at G2/M phase via an association with the cellular protein EDD, a mediator of DNA-damage signal transduction. In the early phase of HHV-6 infection, U14 colocalized with EDD dots in the nucleus, and similar colocalization was also observed in cells transfected with a U14 expression vector. When the carboxyl-terminal region of U14 was deleted, no association of U14 and EDD was observed, and the percentage of cells in G2/M decreased relative to that in cells expressing wild-type U14, indicating that the C-terminal region of U14 and the U14–EDD association are critical for the cell-cycle arrest induced by U14. These results indicate that U14 is a G2/M checkpoint regulator encoded by HHV-6.

## Introduction

Human herpesvirus-6 (HHV-6), a member of the beta herpesvirus family, is classified into two viruses, HHV-6A and HHV-6B [1]. HHV-6B causes exanthem subitum, and persists as a lifelong latent infection [2,3], whereas the pathogenesis of HHV-6A remains unknown. Viral reactivation later in life can lead to severe and even fatal diseases in immune-compromised individuals [4,5]. The genome size of HHV-6 is approximately 160 kbps and encodes about 100 ORFs [6,7,8]. Some of these ORFs are unique to HHV-6, and the functions of some ORFs remain unclear.

Previously, we found that the HHV-6-encoded U14 protein is expressed at an early phase of infection, and is distributed in a dot-like pattern in the nucleus. During the late phase of infection, its localization changes to the cytoplasm mainly, and the protein is incorporated into virions, possibly functioning as a tegument protein [9]. We wished to understand why U14 localizes as nuclear dots in the early phase. Based on this localization and the timing of expression, we speculated that U14 functions in viral replication. Therefore, we tried to identify cellular molecules that associate with U14. We also found that U14 interacts with cellular p53, and that p53 is incorporated into virions along with U14 [9].

In this study, we identified a HECT E3 ubiquitin ligase, E3 identified by differential display (EDD), which associates with U14. Previous work showed that EDD is involved in the regulation of cell proliferation and tumorigenesis [10,11]. Furthermore, EDD plays a role in DNA-damage signaling: EDD binds the DNA-dependent protein kinase-interacting protein CIB1, modulates the activity of CHK2, and is an established mediator of G1/S, intra-S, and G2/M phase checkpoints [11,12,13]. EDD inhibits ataxia telangiectasia mutated (ATM)-mediated phosphorylation of p53, and also plays a role in ensuring smooth G1/S progression [14].

To promote their own replication, viruses control cell-cycle progression [15,16,17,18,19]. HHV-6 infection also induces cell-cycle arrest. For example, infection of T cells with HHV-6 is associated with cell-cycle arrest at the G1/S or G2/M phase [20,21,22,23,24,25], and HHV-6B infection of Molt3 cells causes cell-cycle arrest at the G1 phase concomitant with accumulated and phosphorylated p53 [25]. Inhibition of cell proliferation by viruses can occur through both p53-dependent and-independent pathways [25,26,27]. HHV-6B induces p53 Ser392 phosphorylation by an atypical pathway independent of CK2 and p38 kinases [28]. The cell-cycle arrest induced by HHV-6 in the G2/M phase is accompanied by inhibition of Cdc2-cyclin B1 kinase activity and a significant increase in phosphorylation of Cdc2 at the Tyr15 inhibitory site [23]. HHV-6 alters the E2F1/Rb pathway and E2F1 localization and causes cell-cycle arrest in infected cells [24]. This also causes an elevation in levels of the transcription factor E2F1, which promotes expression of genes important for viral DNA synthesis, such as U27 and U79 [24,29]. During HHV-6B infection, the majority of cellular p53 is inactivated and stabilized in the cytoplasm, most likely due to a reduction in p53 degradation [30]. Recent work showed that HHV-6 DR6 protein has the ability to inhibit the G2/M transition independently of p53 [31]. In addition, HHV-6B U19 inhibits p53-dependent cell death [32].

As described above, several genes encoded by HHV-6 have been shown to contribute to the cell-cycle arrest. Here, we found that U14 protein whose function had been unknown in early phase of HHV-6-infection, induces cell-cycle arrest at G2/M phase via an association with EDD for which the C-terminus of U14 is required. Thus, U14 is a G2/M checkpoint regulator encoded by HHV-6.

## Materials and Methods

### Cells and viruses

Molt3 cells were cultured in RPMI-1640 medium supplemented with 8% fetal bovine serum (FBS). 293T, 293 and HeLa cells were cultured in Dulbecco’s modified Eagle’s medium (DMEM) supplemented with 8% FBS. Umbilical cord blood mononuclear cells (CBMCs) were cultured as described previously [33]. CBMCs were provided by H. Yamada (Kobe University Graduate School of Medicine, Kobe, Japan) and purchased from the Cell Bank of the RIKEN BioResource Center, Tsukuba, Japan. Regarding CBMC usages, the study was approved by the ethics committee of Kobe University Graduate School of Medicine and National Institute of Biomedical Innovation.

The HHV-6A strain U1102 [34] were used for this study, and HHV-6 cell-free virus was prepared as described previously [35]. CBMCs were infected with HHV-6A, cultured in medium with phosphonoformic acid (PFA; 100 μg/ml), which inhibits viral DNA synthesis, and harvested at 24 hpi.

### Antibodies

Monoclonal antibody (Mab) BU14, which recognizes the C-terminal region of HHV-6 U14, was described previously [9]. Mab AgQ1–119, for HHV-6A gQ1 was described previously [36] Anti-HA antibody (clone HA-7; Sigma), rabbit polyclonal antibody against EDD (BETHYLor Abcam), and anti–α-tubulin antibody (Sigma) were purchased from the indicated suppliers. Alexa Fluor 488- or 594-conjugated F(ab’)_2_ fragment of donkey anti-rabbit or-mouse immunoglobulin G (IgG) (Invitrogen) was used as a secondary antibody.

### Immunofluorescence assay

Indirect immunofluorescence assay (IFA) was performed as described previously [33,37,38]. Specific IFA signals were detected using a confocal laser-scanning microscope (Olympus FluoView FV1000; Olympus).

### Western blotting

Western blotting was performed as described previously [37].

### Immunoprecipitation and LC/MS analysis

To investigate protein–protein associations, 293T cells transfected with U14 expressing plasmid were lysed in TNE buffer (10 mM Tris-HCl [pH 7.8], 0.15 M NaCl, 1 mM EDTA, and 1% NP-40 [Nacalai Tesque]). For immunoprecipitations, antibodies were bound to protein G–Sepharose (GE Healthcare) and then cross-linked with protein G using dimethyl pimelimidate (DMP; Thermo Scientific) [39]. Whole-cell extracts were then incubated with the appropriate protein G–Sepharose-bound antibody. Bound proteins were eluted with 0.1 M glycin (PH 2.5) and neutralized by using 1 M Tris-HCl (PH 9.0). The eluates were further prepared for LC/MS analysis as described elsewhere [40,41]. LC/MS analysis was performed using a Qstar-XL mass spectrometer (Applied Biosystems).

### Plasmid construction

N-terminally HA-tagged HHV-6A U14 and its deletion-mutant expression plasmids were constructed using plasmid pCAGGS. pCAGGS was kindly provided by Jun-ichi Miyazaki (Osaka University, Japan) [42]. DNA fragments were amplified by PCR from cDNA from CBMCs infected with the HHV-6A strain U1102, and then inserted in frame into pCAGGS via the *Not*I and *Eco*RI sites.

### Plasmid transfection

293T cells were transfected with expression plasmids using Lipofectamine 2000 (Invitrogen) or the calcium phosphate method, as described previously [43].

### Construction of replication-incompetent recombinant adenovirus vectors

U14 and its mutant genes were excised from each pCAGGS-MCS-expression plasmid using the *Kpn*I and *Not*I sites, and then cloned into the pHMCA5 vector [44]. The resultant plasmids were digested with I-*Ceu*I and PI-*Sce*I, and then cloned into pAdHM34 adenovirus vector [45]. To generate recombinant adenoviruses, 293 cells were transfected with *Pac*I-digested adenovirus vectors using Lipofectamine 2000 [46]. Ten days later, the viruses were released from the cells by freezing and thawing, and then amplified in 293 cells. Recombinant adenoviruses were purified by CsCl gradient ultracentrifugation and titrated using the Adenovirus Titration Kit (Clontech).

### Replication-incompetent recombinant adenovirus infection

HeLa cells were infected with empty adenovirus vector or adenovirus expressing U1102 U14 or its deletion mutants at a multiplicity of infection (MOI) of 100. Infections were carried out for 1 h at 37°C in 95% air/5% CO_2_.

### Cell-cycle analysis by flow cytometry

HeLa cells infected with replication-incompetent recombinant adenovirus were trypsinized and washed. Subsequently, cells were fixed overnight in cold 70% ethanol at-20°C. The fixed cells were washed and then incubated for 30 min at room temperature with PBS containing 20 μg/ml propidium iodide (PI), 0.2 mg/ml RNase A, and 0.1% Triton X-100. Flow-cytometric analysis was performed on a Cell Analyzer EC800 (SONY).

### Statistical analysis

Data are expressed as mean ± standard deviations (SD). Significance between groups was examined using Student’s *t* test. *P* values less than 0.05 were considered to represent statistically significant differences.

## Results

### Identification of cellular molecules that associate with U14

Previously, we showed that p53 interacts with the HHV-6 U14 protein in HHV-6 infected cells and is incorporated into virions during the late phase of infection. Early in HHV-6 infection, U14 localizes in the nucleus, where it is distributed in a dot-like pattern. Subsequently, U14 translocates to the cytoplasm, possibly to function as a tegument protein during the late phase of infection. To elucidate the function of U14 more precisely, we tried to identify other cellular molecules that associate with it. To this end, we first transfected HHV-6B U14-expressing vector or empty vector into 293T cells and immunoprecipitated the protein from lysates using an anti-U14 monoclonal antibody (Mab). For immunoprecipitations (IP), the Mab was bound to protein G–Sepharose and then cross-linked to protein G. Whole-cell extracts were then incubated with the appropriate protein G–Sepharose-bound antibody. Bound proteins were eluted, and the eluates were prepared for LC/MS analysis. p53 and EDD were mainly detected in the eluates of U14-expressing cell lysates.

**Fig 1:**
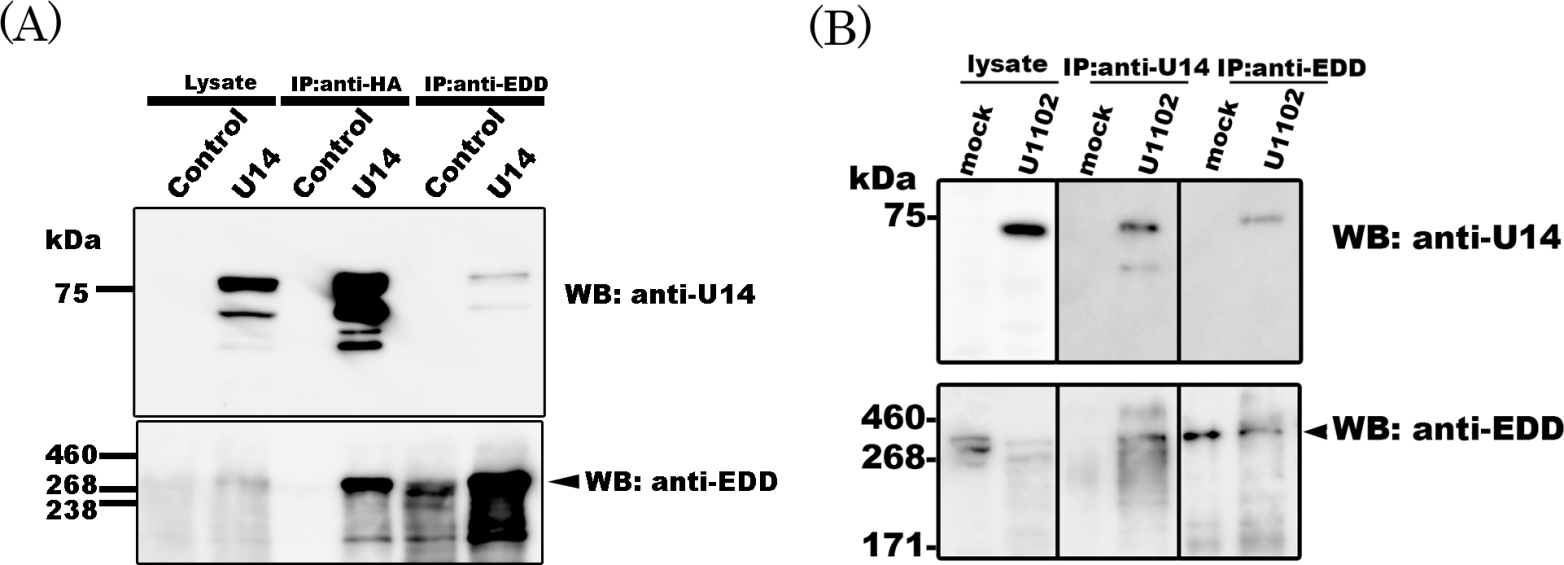
EDD is a cellular molecule that associates with HHV-6 U14. (A)293T cells were transfected with HA-tagged HHV-6A U14 or empty vector (as a control). Transfected cells were lysed at 72 h post-transfection. The lysates were immunoprecipitated (IP) with anti-HA or anti-EDD antibody, followed by Western blotting (WB) with anti-U14 (Mab BU14) or anti-EDD antibody. (B)Mock- or HHV-6A-infected cell lysates were immunoprecipitated with anti-U14 or anti-EDD antibody followed by Western blotting.

To confirm the association between EDD and U14, we transfected HA-tagged U14 protein into 293T cells and immunoprecipitated whole-cell lysates using an anti-HA antibody (to bind tagged U14), anti-U14 Mab, or anti-EDD antibody. The resultant immunoprecipitates were subjected to Western blotting with the anti-U14 Mab or anti-EDD antibody. As shown in Fig 1, EDD co-precipitated with U14, and U14 reciprocally co-precipitated with EDD. Next, the association was examined in lysates of HHV-6A-infected Molt3 cells. As expected, EDD and U14 reciprocally co-precipitated (Fig 1).These results confirmed that U14 associated with EDD, in cells transiently expressing U14 and in cells infected with the HHV-6A virus.

**Fig 2:**
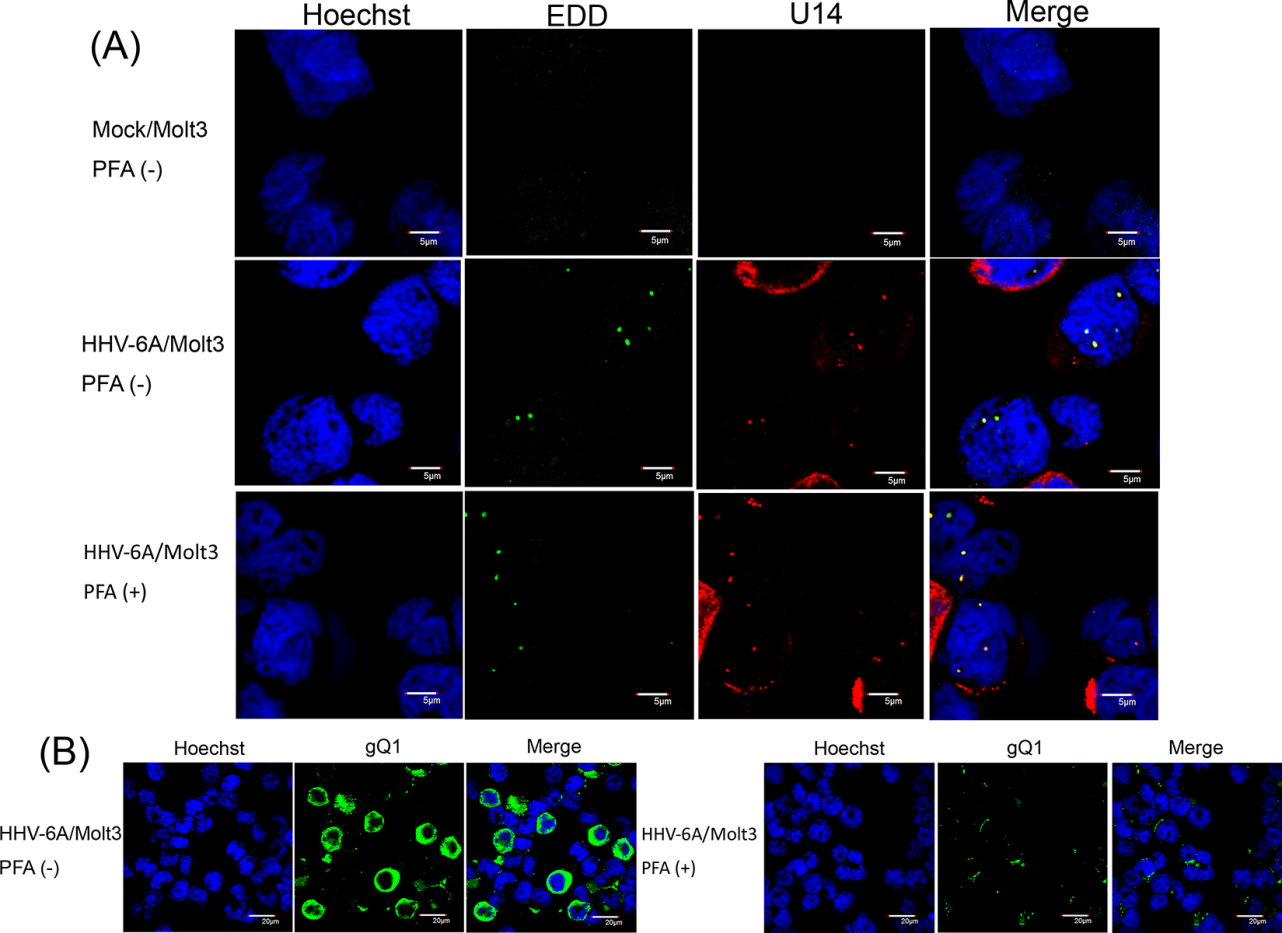
Subcellular localization of U14 and EDD in HHV-6A-infected cells. HHV-6A- or mock-infected Molt3 cells, with or without PFA, were harvested at 24 h post-infection (pi), fixed, and subjected to IFA using antibodies against U14 and EDD (A) or gQ1(B); nuclear DNA was counterstained with Hoechst 33342. Merged panels show colocalized U14 and EDD in nuclei (A). gQ1 was detected in the cells without PFA, but not with PFA (B). Scale bars: 5 μm (A) and 20μm (B). Single sections are shown.

Next, we examined the localization of U14 and EDD in HHV-6A-infected cells. To this end, we infected Molt3 with HHV-6A, cultured the cells with or without phosphonoformic acid (PFA), which inhibits viral DNA synthesis, and fixed the cells at 24 h post-infection (pi). As shown in Fig 2, in the early phase of infection, U14 localized as dots in the nucleus, even in the presence of PFA, as previously reported [9]. EDD also localized in nuclear dots and colocalized with U14 in HHV-6A-infected cells, with or without PFA. By contrast, in mock-infected cells, EDD localized in the nucleus, but as tiny faint dots. These results indicated that, in HHV-6A-infected cells, EDD localizes to prominent nuclear dots.

### Localization and association of U14 and EDD

**Fig 3:**
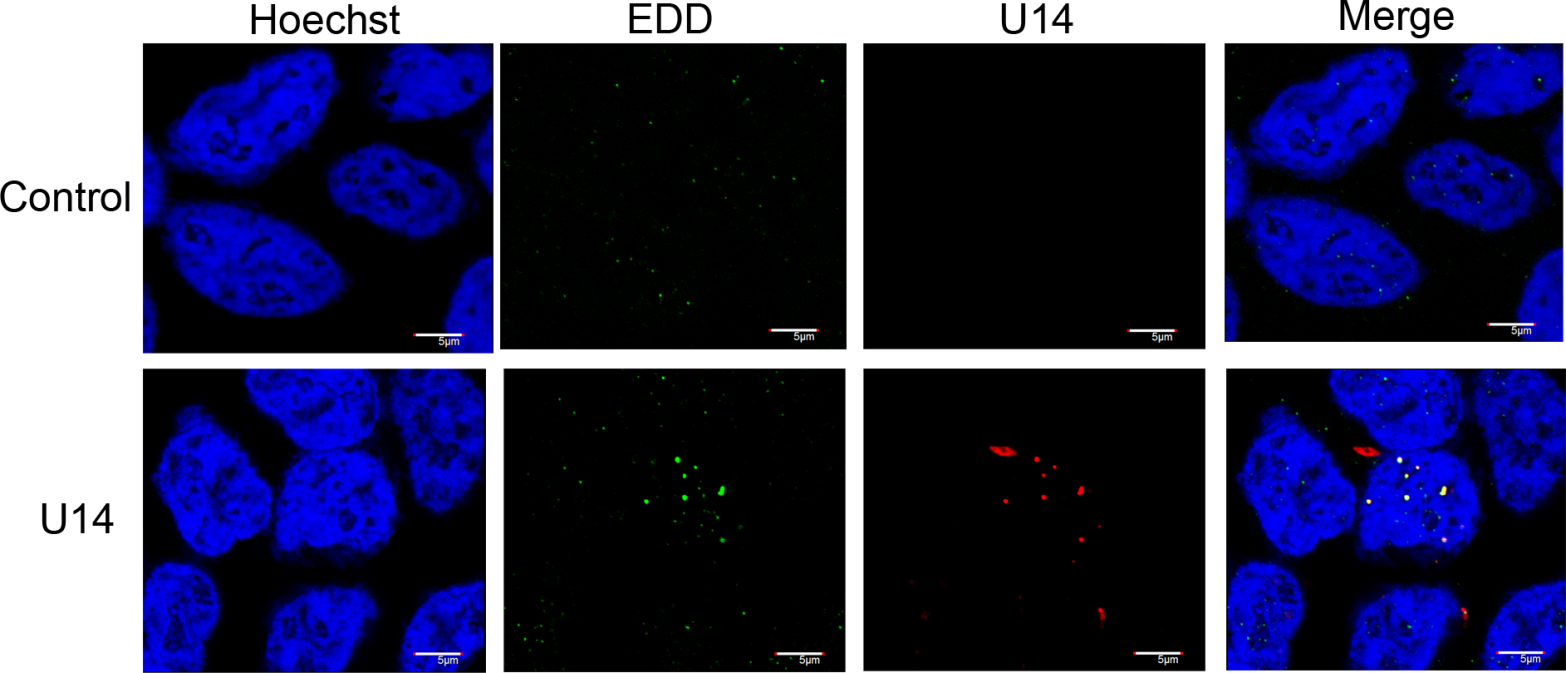
Subcellular localization of U14 and EDD in U14-expressing 293T cells. 293T cells were transfected with HHV-6A U14, its deletion mutants, or empty vector (as a control). Cells were harvested at 12 h post-transfection, fixed, and subjected to IFA using antibodies against HA (for U14) and EDD; nuclear DNA was counterstained with Hoechst 33342. Scale bars: 5 μm. Single sections are shown.

To confirm whether the altered localization of EDD in HHV-6A-infected cells was due only to the association with U14, we transfected the U14 gene into 293T cells and observed the localization of endogenous EDD. At 12 h post-transfection, the cells were fixed and co-stained with anti-EDD and anti-U14 antibodies. As in HHV-6A-infected cells, transfected U14 localized to nuclear dots. In cells transfected with empty vector, EDD appeared only as tiny faint dots (Fig 3, control), but in cells transfected with U14, EDD shifted into larger nuclear dots that colocalized with U14. These results demonstrated that the change of localization of EDD observed in HHV-6A-infected cells was caused by co-expression with U14.

**Fig 4:**
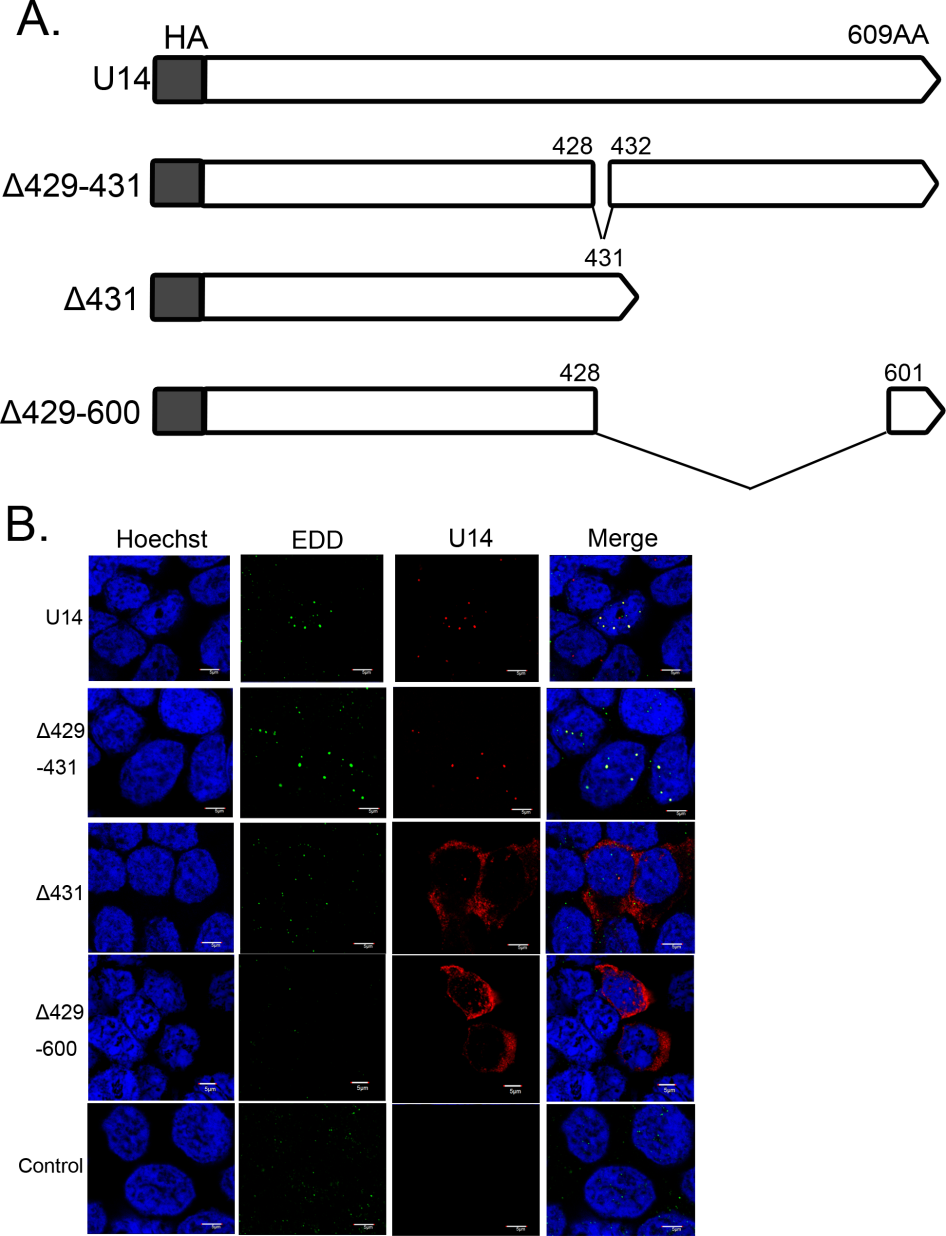
Schematic diagram of HHV-6A U14, its deletion mutants, and the localization of each mutant. (A) The HHV-6A strain U1102 U14 and its deletion mutants (white box) were N-terminally fused to the HA tag (gray box). Numbers indicate positions in the amino acid sequence of U14. (B) 293T cells were transfected with HHV-6A U14, its deletion mutants, or empty vector as a negative control. The cells were harvested at 12 h post-transfection, fixed, and subjected to IFA using antibodies against HA (for U14) and EDD; nuclear DNA was counterstained with Hoechst 33342. Scale bars: 5 μm. Single sections are shown.

To determine whether co-expression of EDD with U14 is sufficient for the dot-like localization of EDD, we constructed several U14 mutants (Fig 4A) and then analyzed the colocalization of EDD with the mutant proteins. 293T cells were transfected with U14 or its mutants; at 12 h post-transfection, cells were fixed and co-stained with anti-U14 and EDD antibodies. Like wild-type U14, U14Δ429–431 colocalized with EDD in nuclear dots, whereas U14Δ431 or U14Δ429–600 localized mainly in the cytoplasm and did not colocalize with EDD. Localization of EDD was not altered in any of these transfected cells.

**Fig 5:**
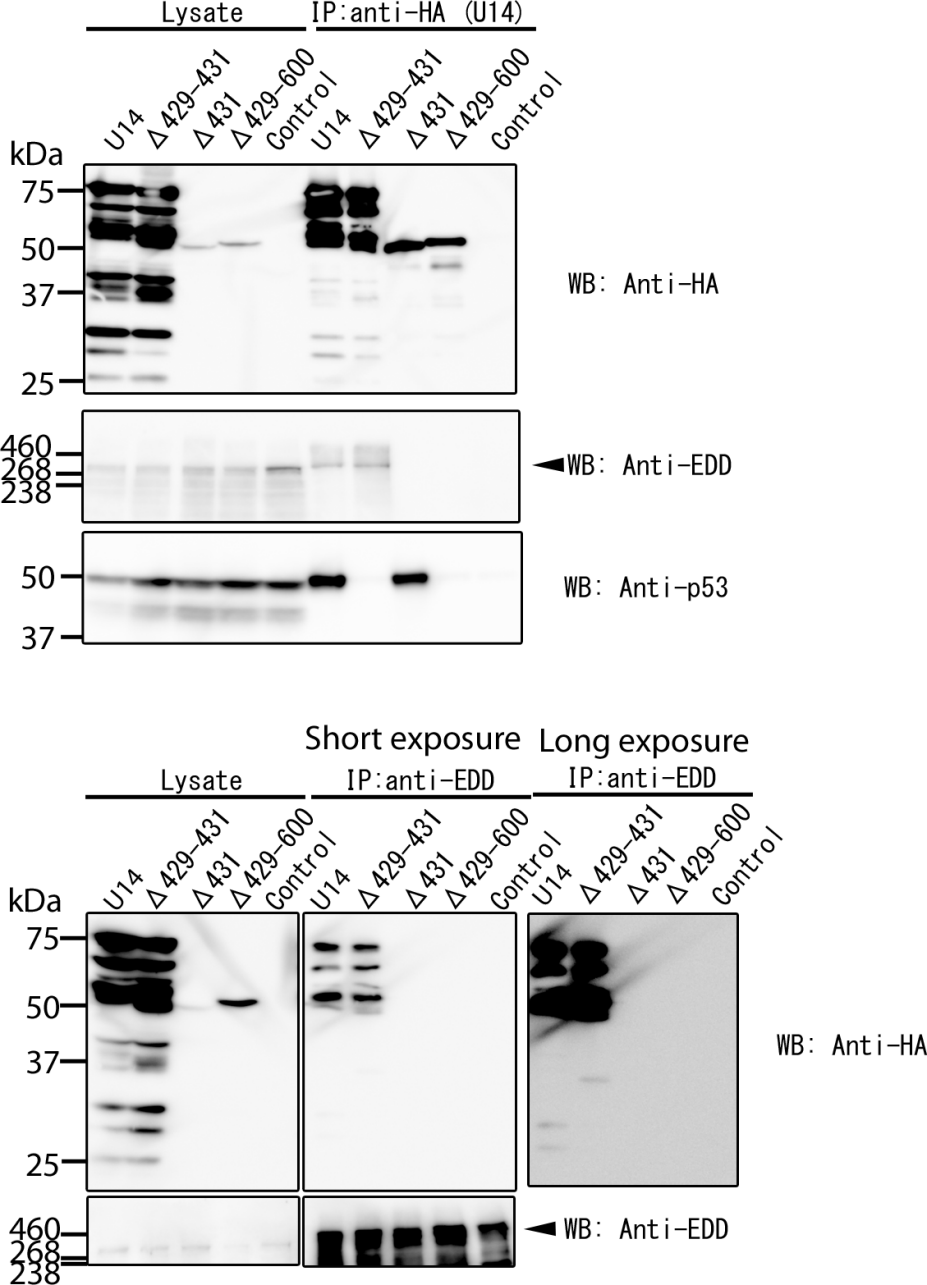
Associations of U14 with EDD and p53. 293T cells were transfected with HA-tagged HHV-6A U14, its deletion mutants, or empty vector (as a negative control). Transfected cells were lysed with TNE buffer at 72 h post-transfection. The lysates were immunoprecipitated (IP) with anti-HA antibody (upper) or anti-EDD antibody (lower), and then analyzed by Western blotting with antibody against HA (to detect U14), EDD, or p53. These data show one of three independent experiments and data in the upper and lower panels were from two independent experiments. WB; Western blotting.

Next, we examined co-precipitation of EDD and each U14 mutant by IP followed by Western blotting. As shown in Fig 5, EDD reciprocally co-precipitated with U14 and U14Δ429–431, but not U14Δ431 or U14Δ429–600. These results also suggested that the association between U14 and EDD determines the colocalization of the two proteins, and that the association of EDD with U14 is sufficient for the dot-like localization of EDD. Although the abundant expression of U14Δ431 and U14Δ429–600 (compared with that of wild-type U14) could only be confirmed with IFA (Fig 4), but not with western blotting (indicated as Lysate in Fig 5), the association of EDD with U14Δ431 or U14Δ429–600 could not be detected even in longer exposure of western blotting (lower right panel in Fig 5). These results show that U14Δ431 and U14Δ429–600 didn’t associate with EDD. In addition, U14Δ429–431 did not co-precipitated with p53, although it did co-precipitate with EDD, whereas U14Δ431 co-precipitated with p53 but not with EDD (Fig 5). Thus, the C-terminus of U14 is crucial for the association with EDD, and the interaction of U14 with p53 does not affect the change of EDD localization.

### U14 induces cell-cycle arrest at G2/M phase

Next, we investigated the functional significance of the U14–EDD association. EDD mediates DNA-damage signal transduction [12], and it is necessary for G1/S and intra-S-phase DNA-damage checkpoint activation and for maintenance of G2/M arrest after double-strand DNA breaks [13]. Therefore, we hypothesized that U14 expressed during the early phase of HHV-6 infection might influence DNA checkpoint activation by associating with EDD in HHV-6-infected cells.

**Fig 6:**
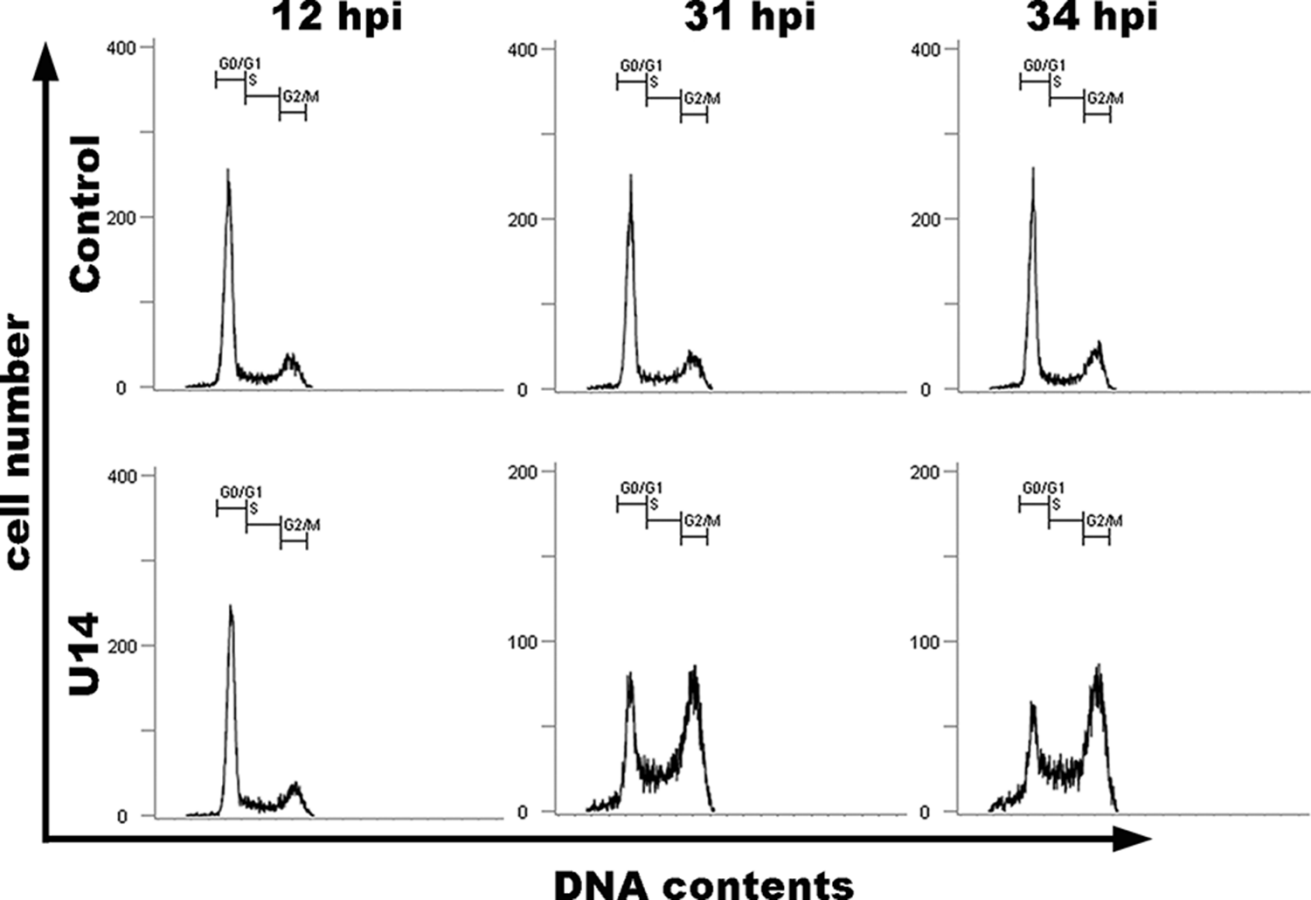
U14 induces cell-cycle arrest in G2/M phase. HeLa cells were empty adenovirus vector-infected (described as control) or infected with adenovirus expressing U14. Subsequently, cells were fixed at the indicated time points and stained with propidium iodide (PI). Flow-cytometric analyses of DNA content were performed by measuring the levels of PI. Numbers in each graph represent percentages of cells in the G0/G1, S, and G2/M phases of the cell cycle. Data represent one of two independent experiments.

**Fig 7:**
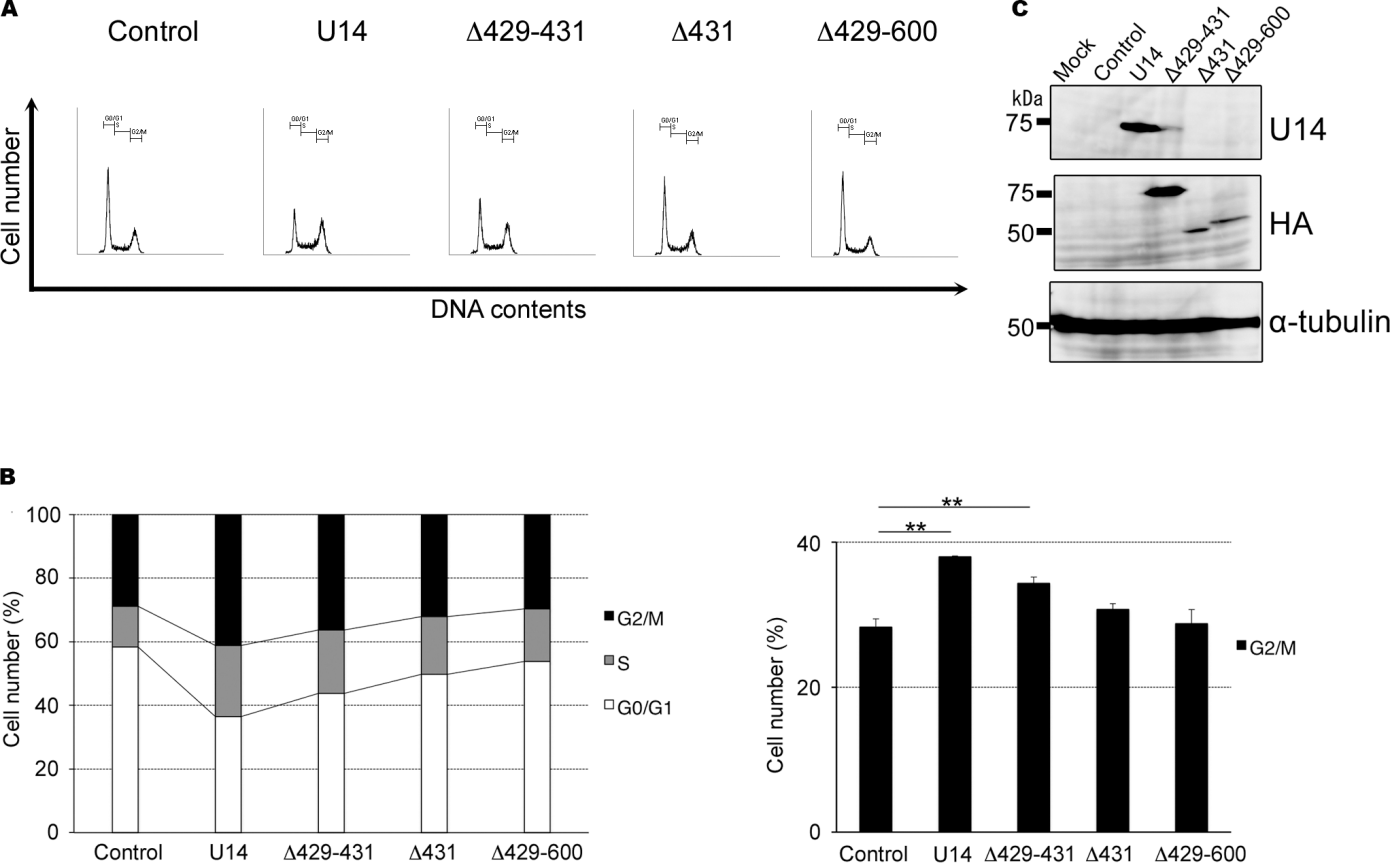
C-terminus of U14 is crucial for cell-cycle arrest in G2/M phase. HeLa cells were infected with empty adenovirus vector (described as control), adenovirus expressing U14, or its deletion mutants (U14Δ429–431, U14Δ431, or U14Δ429–600) at a multiplicity of infection (MOI) of 100. Subsequently (34 hpi), cells were fixed and stained with PI. (A) FACS analyses of DNA content were performed by measuring the levels of PI in U14-expressing cells. Numbers in each graph represent percentages of cells in the G0/G1, S, and G2/M phases of the cell cycle. Data represent one of three independent experiments. (B) The percentages of control or U14-expressing adenovirus-infected cells in G1, S, and G2/M phases at 34 hpi. Data are shown as means ± SD from three independent experiments. Asterisks indicate significant differences (** *p* < 0.01). (C) Cell lysates shown above (collected 34 hpi) were used for Western blotting, and the expression level of each protein was examined.

To study the function of U14 in the cell cycle, we overexpressed U14 in HeLa cells using a replication-incompetent recombinant adenovirus vector. We then performed cell-cycle analyses of empty adenovirus-infected and U14-expressing adenovirus-infected cells at 12, 31 and 34 h post-infection (pi), using propidium iodide (PI) staining to measure DNA content. The empty adenovirus—infected cells were used as control. These analyses revealed that empty adenovirus-infected cells maintained normal cell-cycle profiles during infection, even at 34 hpi (Fig 6). However, U14-expressing cells exhibited a dramatic reduction in the proportion of cells in G1 phase, and a significant increase in the proportion of cells in G2/M phase, relative to empty adenovirus-infected cells. These results show that U14 expression induces cell cycle arrest at G2/M phase, and then induces cell-cycle arrest in G2/M phase. Next, we performed cell-cycle analyses using control-, U14-, U14Δ429–431-, U14Δ431-, and U14Δ429–600-expressing cells (Fig 7). As expected, as shown in Fig 7A and 7B, U14Δ429–431-expressing cells significantly decreased the proportion of cells in G1 phase and increased the proportion of cells in S and G2/M, relative to control cells; however, in U14Δ431- and U14Δ429–600-expressing cells, the reduction in the G1 population was less pronounced, although the G2/M population did increase slightly, indicating that G2/M progression from G1 and S was less strongly induced in U14Δ431- or U14Δ429–600-expressing cells. Expression of U14, U14Δ431, and U14Δ429–600 proteins was confirmed by Western blots performed on the corresponding cell lysates (Fig 7C). These results indicate that the U14 protein can induce cell-cycle arrest in G2/M phase, and that the U14–EDD association plays a key role in this arrest.

## Discussion/Conclusions

Previously, we showed that HHV-6 U14 can interact with p53 [9]. In this study, we identified EDD as a novel U14- associating protein. EDD is an E3 ubiquitin-protein ligase that regulates S phase and G2/M DNA-damage checkpoints, [13]. Furthermore, EDD itself physically interacts with p53, and this interaction blocks phosphorylation of p53 by ATM [14]. EDD inhibits ATM-mediated phosphorylation of p53-Ser^15^ and suppresses the induction of p53 target genes during DNA damage, but this effect does not require its E3 ligase activity. Thus, through binding to p53, EDD inhibits p53 phosphorylation by ATM and plays a role in ensuring smooth G1/S progression [14].

When HHV-6 U14 is expressed in the early phase of infection, it localizes in nuclear dots; later in infection, however, it is localized mainly in the cytoplasm and is incorporated into virus particles as a tegument protein [9]. When EDD is co-expressed with U14, it also localizes in nuclear dots and colocalizes with U14; by contrast, in the absence of U14, EDD appears in the nucleus as fainter and much smaller dots. In HHV-6-infected cells, EDD also appears in prominent nuclear dots containing U14, especially during the early stages of infection. Although U14 translocates to the cytoplasm during the late phase of infection, EDD likely maintains its localization in the nucleus and no longer colocalizes with U14. Based on its localization and association patterns during the early phase of infection, we hypothesized that U14 modulates the cell cycle by associating with EDD. Therefore, we investigated whether U14 could induce cell-cycle arrest using replication-incompetent recombinant adenovirus vector in HeLa cells. As shown in Fig 6, we observed cell-cycle arrest at G2/M phase in U14-expressing cells.

Data obtained using deletion mutants of U14 showed that the association of U14 with EDD enabled cell-cycle into G2/M phase. One deletion mutant of U14, U14Δ429–431, which associated with EDD but not p53, could still induce cell arrest at G2/M phase. Thus, the cell-cycle arrest induced by U14 may not require p53. In addition, neither U14Δ431 nor U14Δ429–600 associated with EDD, although both of them could associate with p53, and both proteins induced cell arrest at G2/M phase much more weakly than wild-type U14. These results indicate that the C-terminal region of U14 is important for the binding with EDD and consequently for U14-induced cell arrest at G2/M phase, and also demonstrate that p53 could not be required for cell-cycle arrest induced by U14. Previously, we showed that U14 could interact with p53 in both HHV-6-infected and U14-expressing cells [9]. Although the role of the U14–p53 interaction remains unclear, it is possible that U14 induces the stabilization and functional inactivation of p53. Consistent with this, HHV-6 can induce cell-cycle arrest at G1/S or G2/M phase [20,21,22,23,24,25], and the HHV-6 DR6 protein has the ability to inhibit G2/M transition independently of p53 [31]; furthermore, HHV-6B U19 inhibits p53-dependent cell death [32]. Therefore, it is possible that U14 promotes cell-cycle arrest at G2/M independently of p53. Taken together, the results of this study demonstrate that U14 acts as an important cell-cycle modulator in HHV-6 infection.
